# Tuning Electronic Properties of the SiC-GeC Bilayer by External Electric Field: A First-Principles Study

**DOI:** 10.3390/mi10050309

**Published:** 2019-05-08

**Authors:** Min Luo, Bin Yu, Yu-e Xu

**Affiliations:** 1 Department of Physics, Shanghai Polytechnic University, Shanghai 201209, China; yubin@sspu.edu.cn; 2 Department of Electronic Engineering, Shanghai Jian Qiao College, Shanghai 201306, China; xuyue@gench.edu.cn; 3 School of Microelectronic, Fudan University, Shanghai 200433, China

**Keywords:** tunable bandgap, SiC/GeC, electric field, first-principles calculation

## Abstract

First-principles calculations were used to investigate the electronic properties of the SiC/GeC nanosheet (the thickness was about 8 Å). With no electric field (E-field), the SiC/GeC nanosheet was shown to have a direct bandgap of 1.90 eV. In the band structure, the valence band of the SiC/GeC nanosheet was mainly made up of C-p, while the conduction band was mainly made up of C-p, Si-p, and Ge-p, respectively. Application of the E-field to the SiC/GeC nanosheet was found to facilitate modulation of the bandgap, regularly reducing it to zero, which was linked to the direction and strength of the E-field. The major bandgap modulation was attributed to the migration of C-p, Si-p, and Ge-p orbitals around the Fermi level. Our conclusions might give some theoretical guidance for the development and application of the SiC/GeC nanosheet.

## 1. Introduction

Research on two-dimensional (2D) materials such as graphene has attracted considerable attention [1,2,3], and has been greatly influential on next-generation electronic and photonic applications due to their rich physical properties and outstanding electronic properties [4,5]. However, graphene is a gapless semiconductor, which causes problems for applications in graphene-based electronic devices. Therefore, many studies have focused on searching for other 2D materials [6,7,8,9]. Recently, except for graphene, research interests have been extended to other similar materials. In recent years, a large number of new 2D materials have been found, such as single-layer MoS_2_ [10,11] and h-BN [12], which exist in wide bandgaps and have attracted much attention.

Considering that the controllable-bandgap engineering of semiconductors is an essential part of nanoelectronics and optoelectronics, a comprehensive investigation on modulating the electronic properties of 2D materials is of great interest and is critical to widening the range of their applications. New 2D materials that consist of similar atomic structures, such as G/BN [13,14], G/MoS_2_ [15], G/SiC [16], have come into our sight, which have aroused intensive studies. If we could control the bandgap of 2D semiconductor materials effectively, there would be new electronic and optical properties, and we could realize the application of these 2D materials in nanoelectronic devices. Very recently, modulation of the bandgap with the help of a geometrical strain or an external electric field has made 2D monolayer sheets particularly interesting materials for device applications at the nanoscale [17,18,19].

Silicon carbide (SiC) and germanium carbide (GeC) are promising two-dimensional materials whose nanostructures have attracted a great deal of attention due to their large bandgaps of 2.6 and 2.1 eV, respectively. This has been verified by many theoretical studies based on DFT calculations [20,21,22,23]. With the development of the semiconductor process, large SiC nanocomposites have been obtained. The energy gap of SiC can vary within wide spectral ranges. Kityk et al. studied the band structures of large SiC nanocomposites both experimentally and theoretically [24,25]. The calculated data agreed with the experimental results. Recently, many studies have been conducted to optimize their electrical properties. Shi et al. studied the electronic properties of the GeC/WS_2_ heterostructure under an electric field (E-field) and modulated its bandgap [26]. Rao et al. studied SiC(GeC)/MoS_2_ heterostructures and found enhanced optical absorption [27] and their results are promising for applications in field-effect transistors. To the best of our knowledge, the studies on the tunable electronic properties of heterostructures containing SiC and GeC are still lacking.

In this paper, we investigate the electronic properties of a SiC/GeC bilayer by using first-principles calculations with van der Waals (vdW) correction. We found that the SiC/GeC bilayer exhibited a direct bandgap at the equilibrium state. Application of the external electric field was found to modulate the bandgap of the heterogeneous bilayers. Under the impact of an E-field, the bandgap, changing from 1.90 to 0 eV, showed a tunable tendency related to the direction and the strength of the E-field. Our results may prove some applications in vdW-based field-effect transistors.

## 2. Materials and Methods

**Figure 1:**
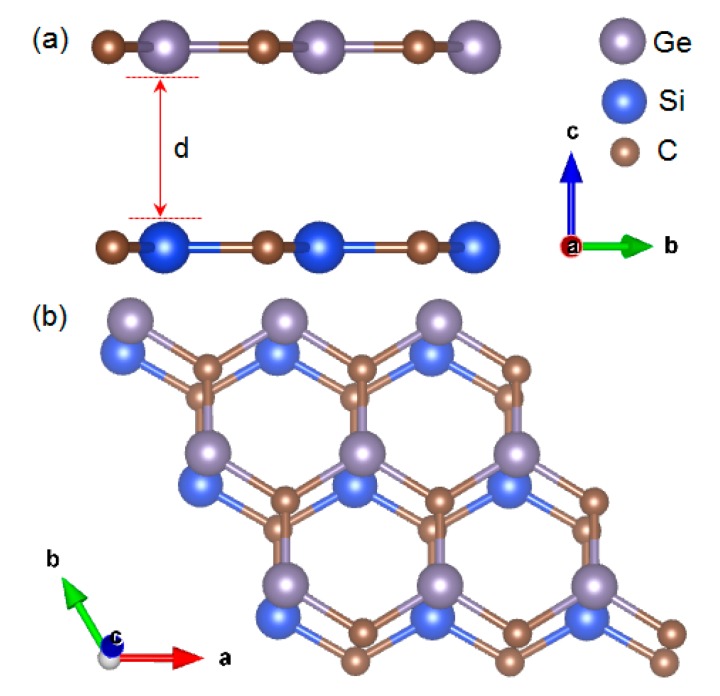
(Color online) (**a**) Side and (**b**) top views of the SiC/GeC van der Waals (vdW) heterostructure. The interlayer distance (d) changes along the *c*-axis.

Electronic structure calculations were performed using the plane-wave-based pseudopotential approach in the framework of density functional theory as implemented in the Vienna Ab initio Simulation Package (VASP) [28]. The electron–ion interaction was described by the projector augmented wave (PAW) method [29], and the Perdew–Burke–Ernzerhof (PBE) generalized gradient approximation [30] was used. The van der Waals corrections (DFT-D2) within the PBE functional proposed by Grimme were also employed [31]. The cut-off energy for the plane-wave basis set was set at 450 eV. The Monkhorst–Pack scheme was used to sample the Brillouin zone with a (5 × 5 × 1) k-mesh. The optimized lattice parameters of SiC and GeC monolayers are 3.09 and 3.23 Å, and the bond lengths of Si-C and Ge-C are 1.79 and 1.86 Å, respectively, which are consistent with other theoretical and experimental results (3.09 and 3.23 Å) [32,33]. The lattice mismatch is about 4.3% between SiC and GeC, which has little effect on the electronic properties of the SiC/GeC heterostructure [34,35]. Thus, we considered a coperiodic lattice consisting of a (4 × 4 × 1) GeC monolayer (16 Ge atoms and 16 C atoms) with (4 × 4 × 1) SiC (16 Si atoms and 16 C atoms), as shown in Figure 1. The vacuum distance of 20 Å was used to reduce the interactions between the periodic images in the supercell model. The atomic positions and the supercell size were fully relaxed, the energy convergent criterion was 10^−6^ eV/atom, and forces on all relaxed atoms were 0.01 eV/Å. All the structures were relaxed by using the PBE function. While we calculated the binding energy, density of states, and bandgaps, we used the DFT-D2 method to describe the van der Waals interaction.

## 3. Results

**Figure 2:**
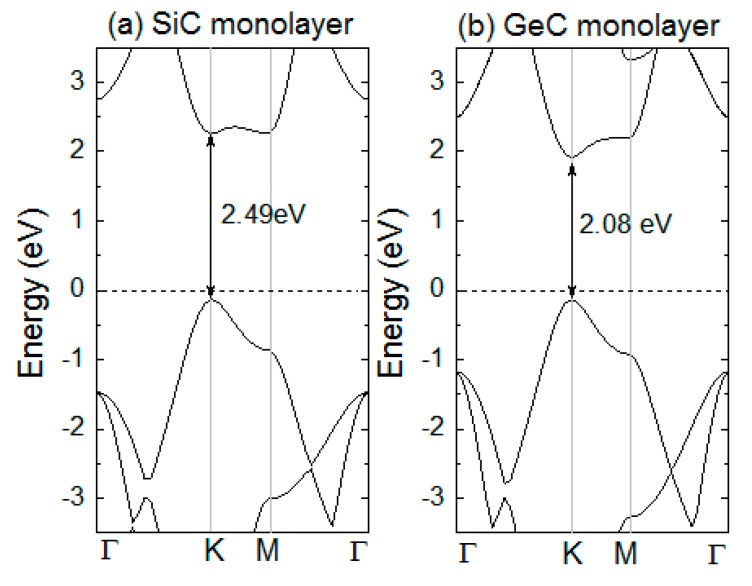
(Color online) Band structures of primitive (**a**) SiC and (**b**) GeC monolayers. The Fermi level is marked by the dashed line.

**Figure 3:**
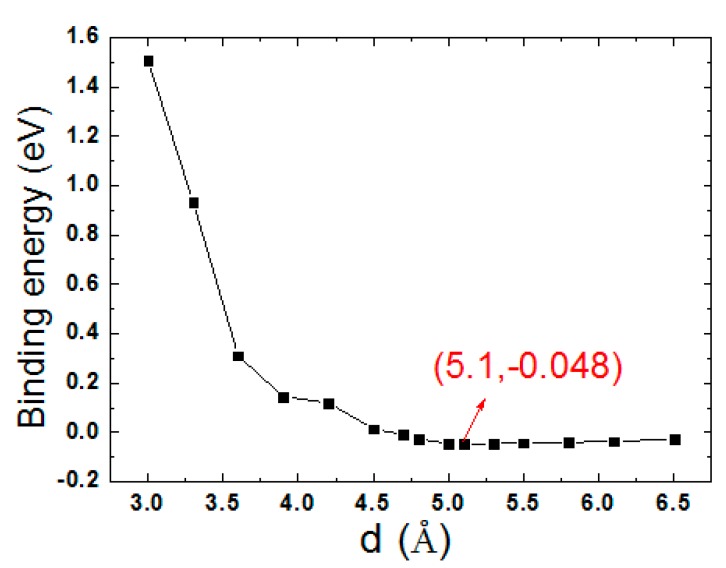
(Color online) Binding energy of the SiC/GeC bilayer as a function of the interlayer distance.

**Figure 4:**
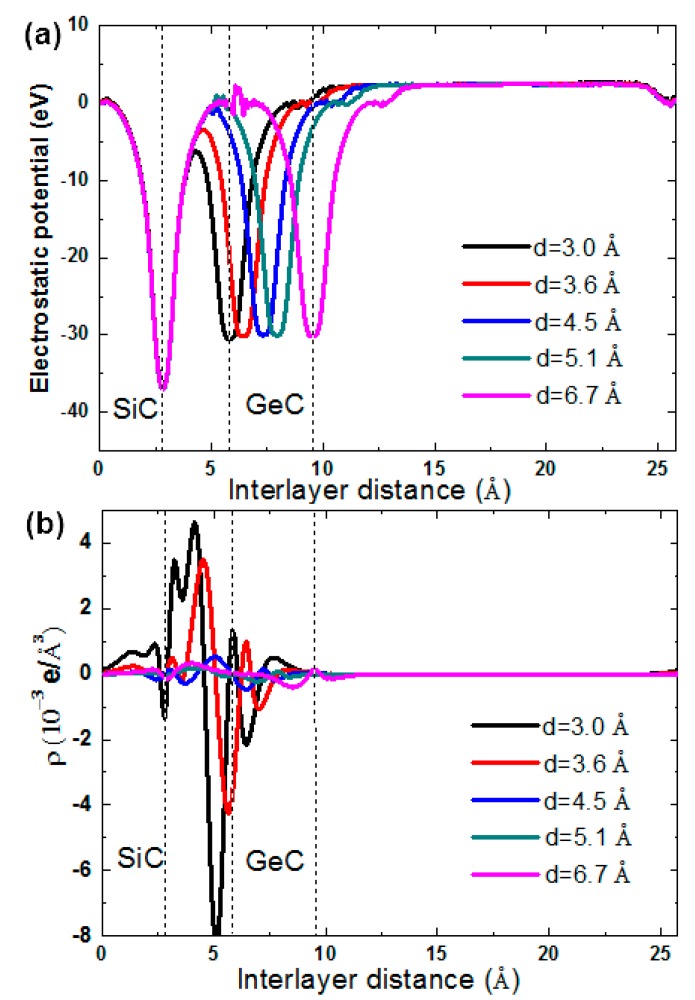
(Color online) (**a**) Electrostatic potentials and (**b**) plane-averaged charge density differences of the SiC/GeC vdW heterostructures with different interlayer distances.

Firstly, we calculated the bandgap of pristine SiC and GeC monolayers. Figure 2a,b shows that the energy gaps of SiC and GeC were 2.49 and 2.08 eV, respectively, which agrees with previous studies [23,36]. As the current bilayer is composed of two different monolayers, the stability of the bilayer is a crucial problem. In order to solve this problem, we calculated the binding energies of the SiC/GeC bilayer, defined as:

E_b_ = E_T_ − (E_SiC_ + E_GeC_),

where E_T_ is the total energy of the bilayer, and E_SiC_ and E_GeC_ are the total energies of SiC and GeC monolayers. From Figure 3 it can been seen that the calculated binding energies changed along with the interlayer distance (d). According to the calculated binding energies, the SiC/GeC bilayer had the lowest value of −0.048 eV at d = 5.10 Å, which indicates that it reached its equilibrium state. To further explore the effects of the interlayer distance on the SiC/GeC bilayer, the plane-averaged charge densities and electrostatic potentials were calculated. From Figure 4a, the potential of the SiC monolayer was much deeper than that of GeC, which seemed to induce a charge shift from the GeC to the SiC monolayer. From Figure 4b, it is clear that more electrons indeed moved from the GeC to the SiC monolayer. These results are in agreement with each other.

**Figure 5:**
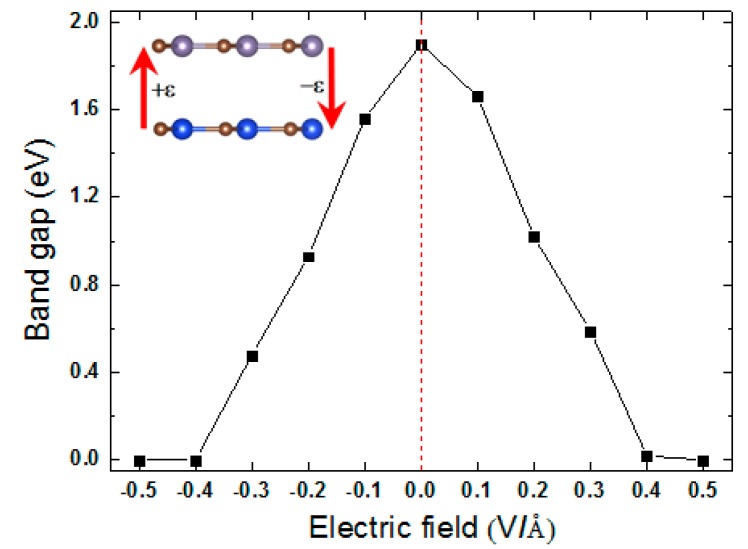
(Color online) Energy bandgap of the SiC/GeC vdW heterostructures as a function of the external electric field.

We now investigate the electronic properties of the bilayer under an E-field. In previous studies, Ni et al. studied the influence of an electric field (from +1.0 to −1.0 V/Å) on the structure of silicene and germanene [37]. Drummond et al. also studied the influence of an electric field (ranging from 0.0 to +0.5 V/Å) on the structure of silicene [38]. According to their results, if a vertical electric field (within limits) is applied to a system, it does not have a significant influence on its structure, but merely causes a redistribution of charges. In this paper, we follow their points and discuss bandgap engineering of the SiC/GeC heterostructure via an external electrical field, ranging from +0.5 to −0.5 V/Å. As shown in Figure 5, two directions were explored. The negative direction of the E-field (−ε) is from the GeC to the SiC monolayer. Accordingly, the opposite direction represents the positive E-field (+ε). Variations of the bandgap with electric fields are presented in Figure 5. For the SiC/GeC bilayer, the bandgap decreased as the strength and direction of E-field changed. An approximate symmetrical tendency appeared between the bandgap and the ε in our calculations. Under the −ε, the bandgap reduced monotonically from 1.90 to 0 eV and disappeared at −0.40 V/Å. When the +ε was applied, similar behavior was observed. The bandgap reduced to zero sharply when the +ε increased from 0 to +0.40 eV. Our results reveal that the E-field could regulate the bandgap of the system effectively. Particularly, the direction of the E-field had the same impact on the bandgap of the system, and the results are slightly different from our previous studies [39].

**Figure 6:**
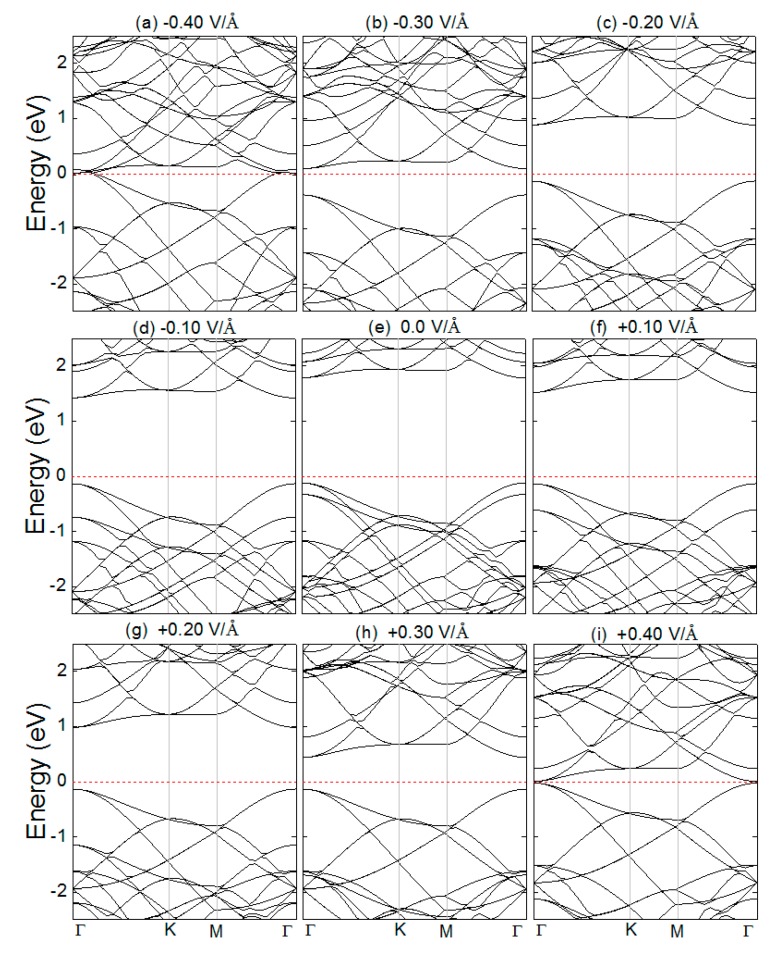
(Color online) The band structures of the SiC/GeC vdW heterostructures with different E-field strength. The Fermi levels are marked by the red dashed line.

**Figure 7:**
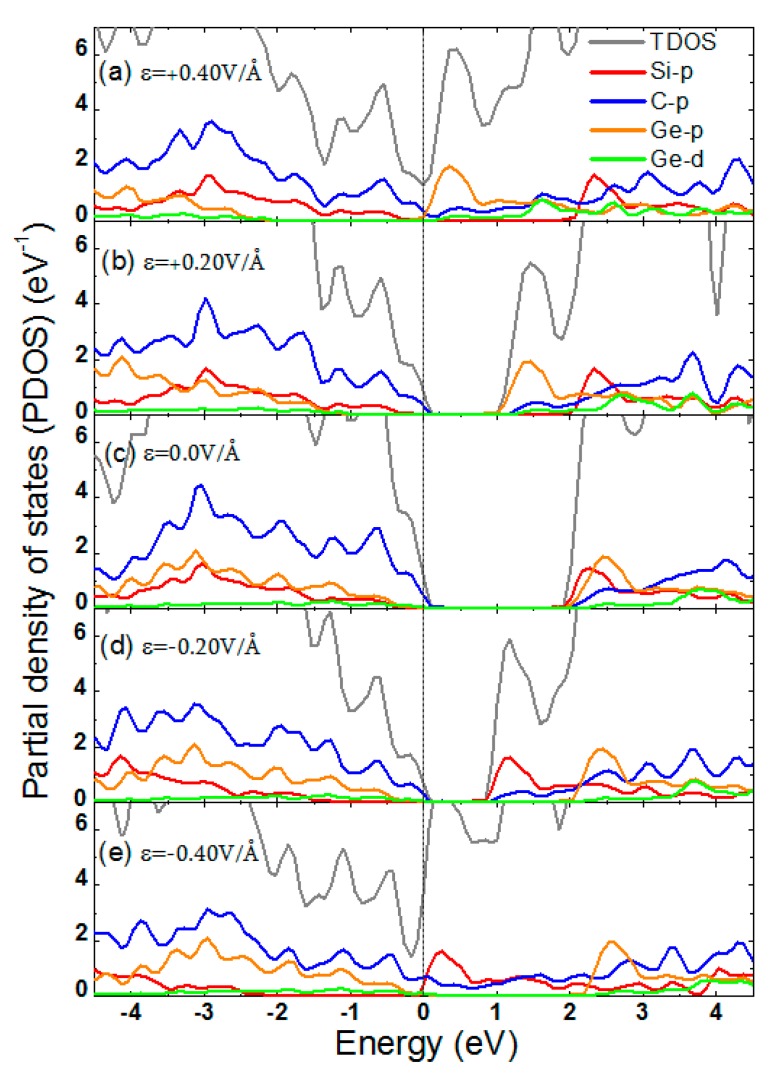
(Color online) Partial densities of states (PDOSs) of the SiC/GeC vdW heterostructures with different E-field strengths. The Fermi level is marked by the black dashed line.

Figure 6 shows the band structures near the Fermi energy (E_F_) of the SiC/GeC bilayer under different E-fields. Under the E-field, the performances of the conduction band (CB) and the valence band (VB) were slightly different. From Figure 6a–d, applying a negative E-field (−ε) resulted in the whole CB moving close to the E_F_. Interestingly, no change was found at the top of the VB. As the strength of the E-field increased, the whole VB ultimately crossed the E_F_, which reduced the bandgap, and then it disappeared. By contrast, under a positive E-field (+ε) very significant differences were seen. As can be seen in Figure 6e–i, the whole VB showed almost no change as the E-field enhanced. However, the situation completely reversed as part of the CB moved close to the E_F_, which led to a decrease of the bandgap. To understand what happens in the band structure, we plotted the partial densities of states (PDOSs), as shown in Figure 7. From Figure 7, we can see the modulations of bandgaps due to the different atomic orbits of C, Si, and Ge. From Figure 7a,b, under the negative E-field (−ε), the states in the bottom of the CB were mainly dominated by C p-orbits, Ge p-orbits, and, partly, Ge d-orbits. States in the top of the VB were mainly dominated by C p-orbits. Under the positive E-field (+ε), as shown in Figure 7d,e, on one hand the p-orbits of Si and C contributed to the modulations in the bottom of the CB, definitively inducing the increasing variations of the bandgap. On the other hand, the states in the top of the VB were dominated by C p-orbits.

Finally, we would like to point out that there are several possible stacking orders of SiC/GeC. In order to gain a comprehensive result that approaches a real situation, all the major stacking geometries should be considered to discover the most stable matching model. In this paper, we only considered one of these, and more work needs to be done in the future.

## 4. Conclusions

In summary, electronic structure calculations were performed to investigate the stability and electronic properties of the SiC/GeC bilayer. We investigated the possible modulation of the bandgap of the SiC/GeC bilayer under the application of an external electric field. The calculation results showed that the SiC/GeC bilayer, in its natural state, had a direct bandgap of about 1.90 eV, and electrons in the SiC–GeC interface preferred to shift from GeC to SiC. Most importantly, the electronic structure could be effectively modulated under the E-field. It was found that the bandgaps of the SiC/GeC bilayer became tunable, and it switched from 1.90 eV to 0 eV as the E-field changed. On the basis of PDOS, such material alterations of the bandgap were due to the migration of different atomic orbits of C, Si, and Ge. Our results indicate that the SiC/GeC bilayer might be a promising candidate for future spintronic device applications.
